# High-cell-density cultivation of *Vibrio natriegens* in a low-chloride chemically defined medium

**DOI:** 10.1007/s00253-023-12799-4

**Published:** 2023-09-23

**Authors:** Richard Biener, Thomas Horn, Alexander Komitakis, Ines Schendel, Leon König, Anna Hauenstein, Alina Ludl, Andrea Speidel, Svenja Schmid, Julian Weißer, Max Broßmann, Sofia Kern, Max Kronmüller, Sonja Vierkorn, Lennart Suckow, Arthur Braun

**Affiliations:** Faculty of Science, Energy and Building Services, University of Applied Sciences Esslingen, Kanalstraße 33, 73728, Esslingen, Germany

**Keywords:** *Vibrio natriegens*, Specific growth rate, Fed-batch, Corrosion, High-cell-density cultivation, Ammonium magnesium phosphate, Citrate, Precipitation

## Abstract

**Abstract:**

*Vibrio natriegens* is a halophilic bacterium with the fastest generation time of non-pathogenic bacteria reported so far. It therefore has high potential as a production strain for biotechnological production processes or other applications in biotechnology. Culture media for *V. natriegens* typically contain high sodium chloride concentrations. The corresponding high chloride concentrations can lead to corrosion processes on metal surfaces in bioreactors. Here we report the development of a low-chloride chemically defined medium for *V. natriegens*. Sodium chloride was completely replaced by the sodium salts disodium hydrogen phosphate, disodium sulfate, and sodium citrate, while keeping the total concentration of sodium ions constant. The use of citrate prevents the occurrence of precipitates, especially of ammonium magnesium phosphate. With this defined medium, high-cell-density fed-batch cultivations in laboratory-scale bioreactors using exponential feeding yielded biomass concentrations of more than 60 g L^−1^.

**Key points:**

- *A defined medium for V. natriegens that only contains traces of chloride was developed*
- *Corrosion processes on metal surfaces in industrial bioreactors can thus be prevented*
- *High yields of biomass can be achieved in fed-batch cultivation with this medium*

**Supplementary Information:**

The online version contains supplementary material available at 10.1007/s00253-023-12799-4.

## Introduction

*Vibrio natriegens* is a marine, facultatively anaerobic, gram-negative proteobacterium (Payne et al. 1961). In complex medium, *V. natriegens* grows in complex medium at 37 °C and neutral pH with a generation time of less than 10 min (Eagon 1962), which is approximately half of the generation time of *Escherichia coli* K12 (Ulitzur 1974). Due to its high growth rates and wide-ranging metabolism, *V. natriegens* has high potential to serve as a host for many biotechnological applications and to significantly accelerate biotechnological processes (Lee et al. 2019; Weinstock et al. 2016; Hoffart et al. 2017; Hoff et al. 2020; Thiele et al. 2021). A genetically engineered *V. natriegens* strain produced alanine under anaerobic conditions from glucose with a space-time yield of 34 g L^−1^ h^−1^, which is about 9 and 13 times higher than space-time yields obtained by genetically engineered *E. coli* and *Corynebacterium glutamicum* alanine producer strains, respectively (Hoffart et al. 2017). Routine molecular biology tasks, such as cloning, require the repeated growth of bacterial cultures. Typically, up to 90% of experimental time is allocated to *E. coli* growth (Lee et al. 2016). The ultrafast growth of *V. natriegens* can facilitate strain optimization by minimizing cycle times during metabolic engineering (Hoff et al. 2020). Furthermore, *V. natriegens* can be used to produce small molecules such as alanine (Hoffart et al. 2017) and succinate (Thoma et al. 2022), proteins (Weinstock et al. 2016; Becker et al. 2019), biopolymers (Dalia et al. 2017), and nanoparticles (Fernández-Llamosas et al. 2017).

Cloning pipelines require low-temperature preservation of bacteria. When stored at low temperatures, such as 4 °C, the culturability of *V. natriegens* declines much faster than that of *E. coli* (Weinstock et al. 2016). This might be a drawback for the use of *V. natriegens* compared to *E. coli* (Wang et al. 2023). The high specific growth rate may also be disadvantageous for bioproduction. Often, the biomass yield of a bacterial culture is optimal at intermediate growth rates. The rapid growth may lead to overflow metabolism and futile metabolic cycles, which reduces the biomass and product yield achieved (Hoff et al. 2020).

Another disadvantage in the cultivation of *V. natriegens* is the required high NaCl concentration in the medium to meet the demand for sodium (Payne et al. 1961). The corresponding high chloride concentration can lead to corrosion processes on the metal surface in bioreactors (Junker 2009; Hoffart et al. 2017). Increasing NaCl fractions might also influence ion-exchange approaches in downstream processing for separating the product from the broth and increase the conductibility of the liquid (Hoffart et al. 2017). Therefore, media with reduced chloride content are of interest.

For industrial applications, cultivation strategies are required to reach high biomass concentrations and therefore to achieve high volumetric productivities of produced biological products. For *E. coli*, high-cell-density fed-batch cultivation has been the focus of many studies (Lee 1996; Riesenberg and Guthke 1999). To be able to reach high-cell densities in *E. coli*, the formation of growth inhibitory overflow metabolites such as acetate has to be minimized. This can be reached by keeping the specific growth rate under a critical value during the fed-batch process using an exponential feeding profile (Riesenberg 1991). For *V. natriegens*, only few fed-batch cultivation processes have been reported. Thiele et al. (2021) described a high-cell-density fed-batch process using a chemically defined medium containing NaCl and reached biomass concentrations of up to 55 g L^−1^ at 30 °C. A temperature reduction from 37 to 30 °C had a positive effect on cell growth (Thiele et al. 2021). Other authors report cell densities below these values (Hoffart et al. 2017; Lee et al. 2019; Becker et al. 2019).

This work describes the development of a defined medium for *V. natriegens* with low-chloride concentration, but sufficient sodium levels. Using this medium, a high-cell-density cultivation strategy with exponential feeding was developed and successfully implemented.

## Materials and methods

### Strain

The microorganism used in this study was *Vibrio natriegens* ATCC 14048 (wild type, DSM 759) from the German collection of microorganisms and cell cultures GmbH (DSMZ, Braunschweig).

### Growth media and culture conditions

*Vibrio natriegens* was preserved as 1.5 ml aliquots in a 20 g L^−1^ glycerol solution in LB medium at −80 °C. These frozen stock cultures were used for inoculum preparation. The bacteria were first cultivated in a preculture with complex medium. For this purpose, 250 μL bacterial suspension containing *V. natriegens* was taken from a cryo vial and added to 75 ml modified LB medium with increased NaCl concentration (10 g L^−1^ tryptone, 5 g L^−1^ yeast extract, 20 g L^−1^ NaCl, pH 7) in a 500 ml shake flask with 4 baffles and dissolved oxygen (DO) sensor spots (PreSens Precision Sensing GmbH, Regensburg, Germany). The inoculated preculture was cultured at 30 °C and a shaking frequency of 200 rpm for 6–7 h in a Multitron shaking incubator (Infors HT, Bottmingen, Switzerland) using the Shake Flask Reader SFR from PreSens for online measuring of DO.

This preculture was used to inoculate the shake flasks used for the medium development. The standard medium used as the starting medium for media development is a modified M9 medium supplemented with 20 g L^−1^ NaCl. The medium was adapted in preliminary tests and is referred to here as standard medium VN1. It contains the following composition:

NaCl (20 g L^−1^); glucose (12 g L^−1^); Na_2_HPO_4_ × 2 H_2_O (8.5 g L^−1^); KH_2_PO_4_ (3 g L^−1^); NH_4_Cl (1 g L^−1^); MgSO_4_ × 7 H_2_O (0.74 g L^−1^); (NH_4_)_2_SO_4_ (5 g L^−1^); 1% (v v^−1^) trace element solution.

The trace element solution was taken from Keilhauer et al. (1993) with EDTA added: Na_2_-EDTA × 2 H_2_O (6 g L^−1^); FeSO_4_ × 7 H_2_O (1.64 g L^−1^); CaCl_2_ × 2 H_2_O (1.35 g L^−1^); MnSO_4_ × H_2_O (1 g L^−1^); ZnSO_4_ × 7 H_2_O (0.1 g L^−1^); CuSO_4_ × 5 H_2_O (0.03 g L^−1^); NiCl_2_ × 6 H_2_O (0.002 g L^−1^).

During the media optimization described in the “Results” section, NaCl was replaced with the sodium salts Na_2_HPO_4_, Na_2_SO_4_, and Na_3_-citrate. In the first part of the medium optimization, NaCl was replaced by Na_2_HPO_4_ and Na_2_SO_4_ in different compositions according to Table 1. The second part consisted of replacing NaCl with Na_2_HPO_4_, Na_2_SO_4_, and Na_3_-citrate in different compositions as shown in Table 2. For experiments in shaking flasks, the medium was additionally supplied with 42 g L^−1^ 3-(N-morpholino) propanesulfonic acid (MOPS) to keep the pH as constant as possible. The pH was adjusted to 7.0 by adding 3 M KOH or 2 M H_3_PO_4_.

**Table 1:** Composition of nutrient media for media optimization. Part 1: NaCl in standard medium VN1 was replaced by Na_2_HPO_4_ and Na_2_SO_4_ in different compositions. All other concentrations of media components in medium VN1 remain unchanged. The Na_2_HPO_4_ × 2 H_2_O values indicate the amount added in addition to the 8.5 g L^−1^ in the standard medium VN1. For example, the total concentration of Na_2_HPO_4_ × 2 H_2_O in medium VN4 is 8.5 + 24.3 = 32.8 g L^−1^

		VN1	VN2	VN3	VN4
NaCl	g L^−1^	20	0	0	0
Na_2_SO_4_	g L^−1^	0	24.30	12.15	0
Na_2_HPO_4_ × 2 H_2_O	g L^−1^	0	0.00	12.15	24.30

**Table 2:** Composition of nutrient media for media optimization. Part 2: NaCl in standard medium VN1 was replaced by Na_2_HPO_4_, Na_2_SO_4_, and Na_3_-citrate in different compositions. All other concentrations of media components in medium VN1 remain unchanged. The Na_2_HPO_4_ × 2 H_2_O values indicate the amount added in addition to the 8.5 g L^−1^ in the standard medium VN1. For example, the total concentration of Na_2_HPO_4_ × 2 H_2_O in medium VN6 is 8.5 + 8.09 = 16.59 g L^−1^

		VN5	VN6	VN7	VN8	VN9
		Na_2_HPO_4_ : Na_2_SO_4_ : Na_3_-citrate				
		42:42:16	33:33:33	25:25:50	16.7:16.7:66.7	1 g L^−1^ EDTA
Na_2_SO_4_	g L^−1^	10.21	8.09	6.08	4.05	12.15
Na_2_HPO_4_ × 2 H_2_O	g L^−1^	10.21	8.09	6.08	4.05	12.15
Na_3_-citrate × 2 H_2_O	g L^−1^	4.73	9.84	14.78	19.71	0.00

For media optimization, the bacteria were cultivated in 500 ml shake flasks (4 baffles) with DO sensor spots in the ShakeFlask Reader SFR. The shake flasks were filled with different defined media according to Tables 1 and 2. Every experiment was performed at least in triplicate. Each flask was then inoculated with the preculture to a starting OD600 of 0.5. The flasks were cultured in a Multitron shaking incubator at 30 °C for 7–8 h. To avoid oxygen limitation, the shaking frequency was gradually increased from 200 to 350 rpm guided by the online measured O_2_ concentration. At the end of the experiments, 1 ml samples were taken from each flask, and OD, osmolality, pH, and glucose concentration were measured.

### Fed-batch runs in lab-scale bioreactors

The optimized media in Table 3 were used for the fed-batch cultivations. After glucose depletion, a concentrated feed solution was fed to the bioreactor using an exponential feeding strategy described below. The feed solution had the following composition:

**Table 3:** Composition of the optimized chemically defined media VN6, VN10, VN11, and VN12

	VN6	VN10	VN11	VN12
	g L^−1^	g L^−1^	g L^−1^	g L^−1^
Glucose (shake flask preculture)	12.00	12.00	12.00	12.00
Glucose (Multifors main culture)	24.00	24.00	24.00	24.00
KH_2_PO_4_	3.00	3.00	3.00	3.00
Na_2_HPO_4_ × 2 H_2_O	16.59	16.59	18.63	18.63
Na_2_SO_4_	8.10	8.10	10.13	10.13
Na_3_-citrate × 2 H2O	9.84	9.84	12.3	12.3
NH_4_Cl	1	0	1	0
(NH_4_)_2_SO_4_	5	6.30	5	6.30
MgSO_4_ × 7 H_2_O	0.74	0.74	0.74	0.74
Trace elements (shake flask preculture)	1% (v v^−1^)	1% (v v^−1^)	1% (v v^−1^)	1% (v v^−1^)
Trace elements (Multifors main culture)	2% (v v^−1^)	2% (v v^−1^)	2% (v v^−1^)	2% (v v^−1^)

Glucose (540 g L^−1^); K_2_HPO_4_ (60 g L^−1^); Na_3_-citrate × 2 H_2_O (24 g L^−1^); MgSO_4_ × 7 H_2_O (14.8 g L^−1^); Na_2_-EDTA × 2 H_2_O (0.54 g L^−1^); FeSO_4_ × 7 H_2_O (0.148 g L^−1^); CaCl_2_ × 2 H_2_O (0.122 g L^−1^); MnSO_4_ × H_2_O (90 mg L^−1^); ZnSO_4_ × 7 H_2_O (9 mg L^−1^); CuSO_4_ × 5 H_2_O (2.7 mg L^−1^); NiCl_2_ × 6 H_2_O (0.2 mg L^−1^).

The pH value was adjusted to 6.5.

### Bioreactor setup

For the fed-batch cultivations, two parallel benchtop bioreactors were used (Model Multifors 2, Infors HT, Bottmingen, Switzerland). The maximum working volume is 1 L. Agitation was achieved through three 6-blade Rushton turbine impellers. The temperature was set to 30 °C and the pH value was controlled at 6.75 (± 0.05) using 25% ammonia water and 2 M phosphoric acid. Antifoam (Antifoam 204, Sigma-Aldrich) was fed when required. DO was controlled by a multivariate controller such that the DO value was always above 25% air saturation as described in Biener et al. (2010). The manipulated variables were stirrer speed, air flow rate, and O_2_-content in aeration, respectively. Air flow rates were controlled by thermal mass flow controllers (Red-y, Vögtlin, Aesch, Switzerland).

The bioreactors were filled with 500 ml medium and inoculated using a preculture with the same defined medium to an initial OD600 of 1.7 ± 0.2. Feeding was started after glucose depletion, which was indicated by a sharp increase in DO. The feed solution was fed exponentially according to Eq. 1. The set point of the specific growth rate *μ*_*S*_ was 0.3 h^−1^.

1 $$F_{zu}(t)=\left(\frac{\mu_S}{Y_{X/S}}+m_S\right)\cdot\frac{V_0\cdot c_{X,0}}{c_{S,zu}}\cdot e^{\mu_s\cdot t}$$

The biomass concentration at feeding start (*c*_*X*, 0_) was estimated from a previous correlation of OD600 with cell dry weight (CDW) values and the corresponding volume *V*_0_ was 0.55 L. The biomass/glucose yield coefficient *Y*_*X*/*S*_ was 0.4 grams biomass per gram glucose (gg^−1^) and the maintenance *m*_*S*_ was 0.1 grams biomass per gram glucose and hour (gg^−1^ h^−1^). Glucose feed concentration was 540 g L^−1^. The exponential feed rate was controlled gravimetrically.

### Analyses

During the fed-batch cultivations, samples were taken every 60 min manually. Optical density was measured by an Eppendorf BioPhotometer at 600 nm after proper dilution with 0.9% NaCl solution. CDWs were determined by filling reaction tubes with 2-ml samples. The samples were centrifuged at 16,000 *g* for 10 min and the pellet was washed under the same conditions with 0.9% NaCl before it was dried until constancy of weight was achieved.

For analyses of supernatant components, samples were inactivated at 80 °C for 10 min and centrifuged at 16,000 *g* for 10 min. Enzyme test kits from R-Biopharm, Darmstadt were used for the determination of ammonia and acetate in supernatants from culture samples. The concentration of phosphate was measured using a colorimetric assay (Merck, Darmstadt, Germany). Glucose concentration was determined enzymatically using BIOSEN C-line (EKF-diagnostic GmbH, Barleben, Germany). Osmolality was measured using the freezing point osmometer Osmomat 3000 (Gonotec GmbH, Berlin, Germany).

## Results

### Development of a sodium chloride free medium

The main objective of the medium optimization was to replace NaCl in the standard medium with other sodium salts in order to prevent chloride-associated corrosion processes in bioreactors.

In the first series of experiments, NaCl was replaced by Na_2_HPO_4_ and Na_2_SO_4_ in different compositions according to Table 1. In order not to change the supply of sodium ions, the total concentration of sodium ions was kept constant. With these media, cultivations were performed in 500 ml shake flasks. MOPS buffer was added to keep the pH as constant as possible. OD600, glucose concentration, osmolality, and pH were measured at the end of the cultivations. In all cases, glucose was not detectable, indicating that growth was limited by glucose.

**Fig. 1:**
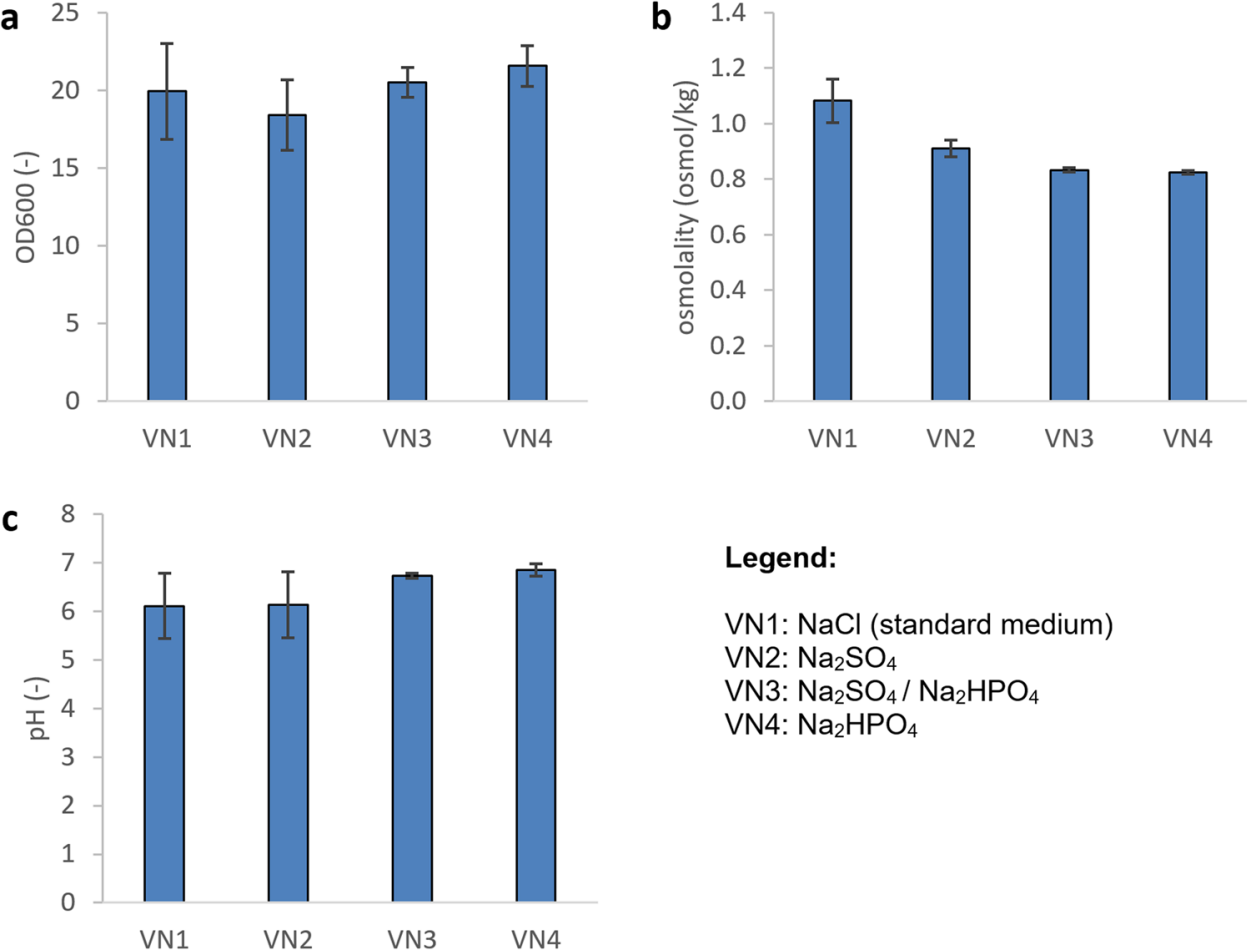
Shake flask cultivations of *V. natriegens* in different media according to Table 1. Values for OD600 (**a**), osmolality (**b**), and pH (**c**) at the end of the cultivation. For each condition, three independent experiments were performed. Error bars show standard deviations

Figure 1 shows the results of these experiments. The final OD600 values show that *V. natriegens* grow similar in all media used. The highest OD600 value of 21.6 ± 1.3 was obtained in medium VN4 with Na_2_HPO_4_ without the use of Na_2_SO_4_. Medium VN2 with NaCl replaced by Na_2_SO_4_ had the lowest OD600 value (18.4 ± 2.3). The results show that NaCl can be replaced completely by other sodium salts.

The osmolalities in the new media at the end of the cultivations were between 0.82 and 0.91 Osmol kg^−1^, which was lower compared to the standard medium VN1 with NaCl (1.08 Osmol kg^−1^). The final pH values were in the range of 6.1 to 6.8.

**Fig. 2:**
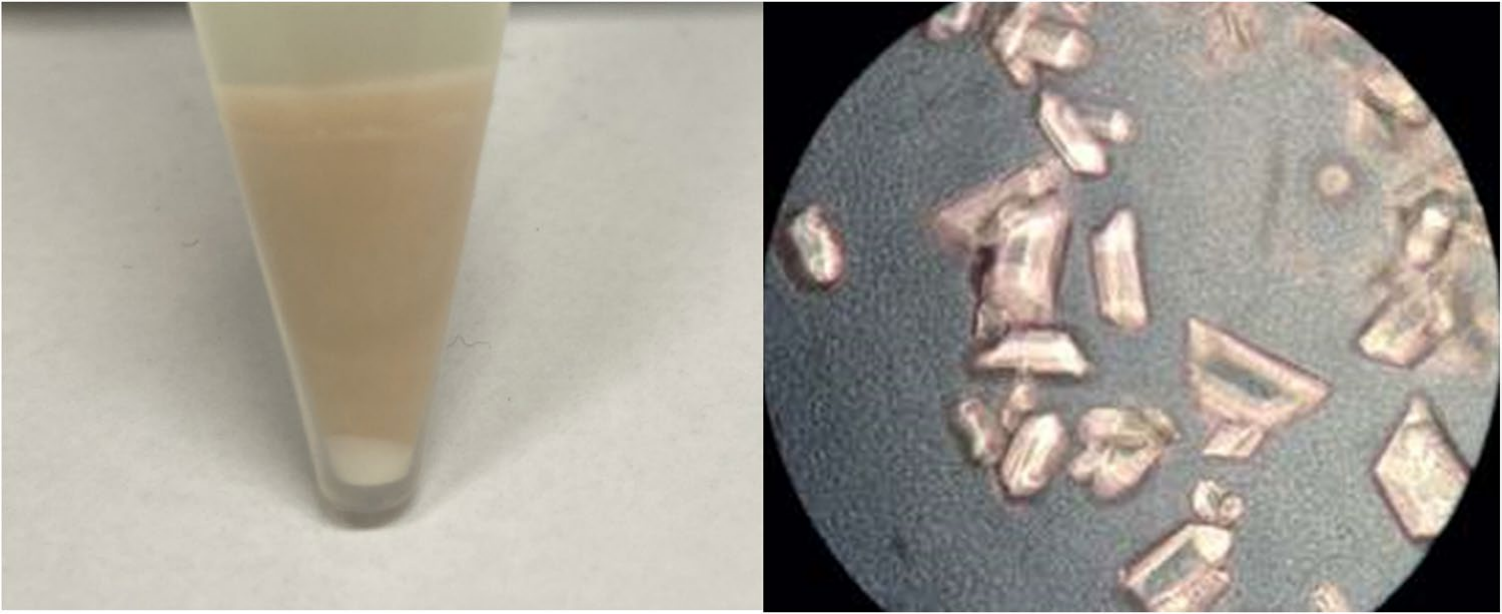
Precipitations in the centrifuged sample and microscopic image of precipitated crystals

The fermentation broth was also visually examined for precipitate formation before and at the end of cultivation. Before the start of the cultivations, no precipitates were visible in the media. In some cases, the samples showed precipitations at the end of the cultivations. Precipitates were clearly visible as a white precipitate below the cell pellet in the centrifuged sample (see Fig. 2).

Microscopic examination of these deposits indicates precipitation of ammonium magnesium phosphate (AMP) due to the characteristic crystals (see Fig. 2). AMP crystallizes in the orthorhombic system as white to yellowish or brownish-white pyramidal coffin-like crystals (Whitaker and Jeffery 1970).

In order to specifically investigate precipitation phenomena in the new media, the influence of pH on medium VN4 (NaCl replaced by Na_2_HPO_4_) at room temperature of 20 °C was first examined without cells. Since precipitation can also occur several hours after preparation of the solutions (Pirt 1975), the solution was kept at 20 °C for 24 h after preparation and then tested again for precipitation. The MgSO_4_ stock solution was added as the last component and just before that, the pH was adjusted to the respective value. Table 4 summarizes the results obtained. No precipitates were observed at pH 6.5. At pH 6.75 and 7.0, no precipitates were visible at the beginning, but precipitates appeared after 1 day. At pH above 7, precipitation occurred immediately after the addition of MgSO_4_.

**Table 4:** Precipitations of media VN4 (NaCl replaced by Na_2_HPO_4_) depending on the pH value

pH	6.5	6.75	7	7.6	8.3
Precipitations directly after addition of MgSO_4_	None	None	None	+	++
Precipitations after 1 day	None	+	+	++	++

### Cultivations in 1 L bioreactor: influence of pH on growth behavior

**Fig. 3:**
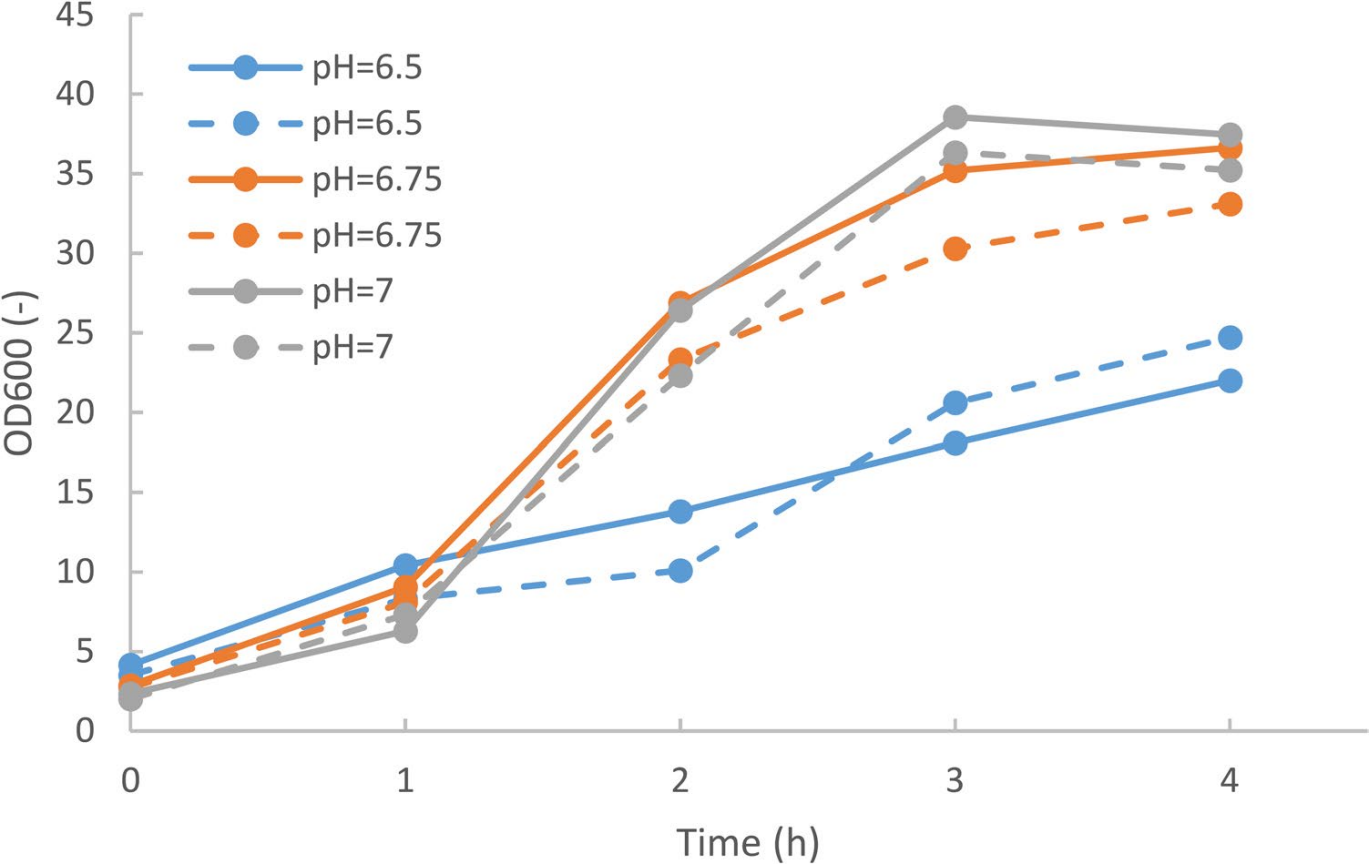
Time profiles of OD600 during cultivations of *V. natriegens* in medium VN3 at different pH values

The precipitation experiments show that the pH value should be kept as low as possible to avoid precipitation. Therefore, the influence of pH on the growth behavior of *V. natriegens* was further investigated. Since it is not possible to precisely control the pH in shake flasks, batch cultivations were performed in 1 L Multifors bioreactors at 30 °C and at pH set points of 6.5, 6.75, and 7.0, respectively. For each pH value, two cultivations were performed. To further reduce the potential for precipitation, medium VN3 with a lower phosphate concentration was used, resulting in similar OD yields of 20.5 ± 0.97 compared to medium VN4 with 21.6 ± 1.3 (see Fig. 1). In these experiments, the glucose concentration was increased to 24 g L^−1^. After 4 h of cultivation, the OD values at pH 6.75 and 7.0 ranged from 33.1 to 37.4, while at pH 6.5, the OD values were much lower at 22 and 24.7, respectively (see Fig. 3).

Therefore, the pH value should be set to 6.75 or above. However, the results above show that precipitation may occur at these pH values.

### Further media optimizations using sodium citrate

The results so far show that NaCl can be completely replaced by Na_2_HPO_4_ and Na_2_SO_4_ with comparable biomass yields. To avoid precipitation in medium VN4, the pH should be adjusted to values below or equal 6.5. However, bacterial growth is reduced at pH values below 6.75. Therefore, it is desirable to further modify the medium to substantially avoid the occurrence of precipitation. This is particularly important for fed-batch cultivations, as critical concentrations for precipitation could be reached by adding media components.

The formation of precipitates can possibly be prevented by the use of complexing agents. For example, EDTA can complex magnesium ions and thereby inhibit the formation of ammonium magnesium phosphate (Das et al. 2017). Citrate can also complex magnesium ions and may contribute to inhibition of AMP formation (Tate et al. 1965; Prywer et al. 2015).

In order to investigate the influence of citrate on the growth behavior of *V. natriegens* and on the occurrence of precipitates, sodium citrate was used as a further sodium salt in addition to Na_2_HPO_4_ and Na_2_SO_4_ for elimination of NaCl in the standard medium VN1. For this purpose, another set of experiments with different mixtures of these three sodium salts according to Table 2 (VN5 to VN8) was carried out in shake flask experiments in the SFR at pH 7.0. Again, the total concentration of sodium ions was kept constant in order to have no change in the supply of sodium ions. In another experiment, the medium was supplemented with EDTA instead of sodium citrate (medium VN9 in Table 2). The occurrence of precipitates at the beginning and at the end of the cultivations was examined.

**Fig. 4:**
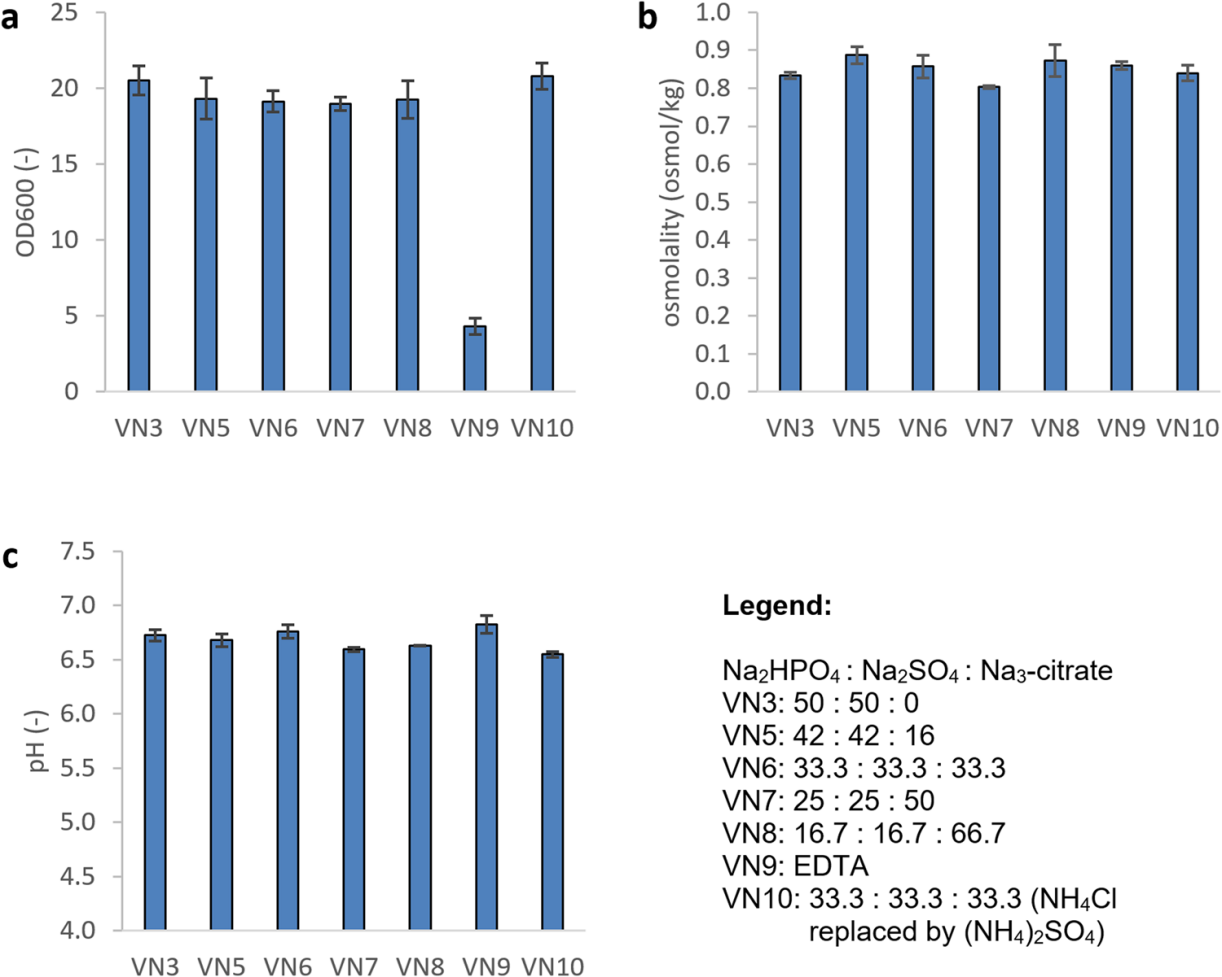
Shake flask cultivations of *V. natriegens* in different media according to Tables 2 and 3. OD600 (**a**), osmolality (**b**), and pH (**c**) at the end of the cultivation. For each condition, three independent experiments were performed. Error bars show standard deviations

The results in Fig. 4 show that in the EDTA medium, VN9 growth was strongly inhibited. All cultivations with sodium citrate (media VN5 to VN8) grew comparably, with mean OD values ranging from 18.9 to 19.3. In runs with medium VN3, slightly higher OD values of 20.5 ± 0.97 were obtained. The osmolalities at the end of the cultivations were between 0.8 and 0.89 Osmol kg^−1^. The final pH values were in the range of 6.6 to 6.83 and glucose was not detectable.

In all media, no precipitations were detected before inoculation and at the end of the runs. Precipitation was practically prevented in all media with sodium citrate. Media VN5 through VN8 were stored sterile without cells at room temperature for another 7 days. Slight precipitation occurred in medium VN5 with the lowest citrate content, while all other media remained precipitation-free. A good compromise between high biomass yield and elimination of precipitation is medium VN6. In a final step to optimize the medium VN6, the existing NH_4_Cl was replaced by (NH_4_)_2_SO_4_ to further reduce the chloride content. This medium is referred to here as VN10. The composition of the optimized growth media VN6 and VN10 is summarized in Table 3. The OD values obtained in medium VN10 were 20.8 ± 0.87 and therefore slightly higher compared to those of media VN5 to VN8 (see Fig. 4). The media VN6 and VN10 were also used for the fed-batch cultures in the Multifors bioreactors. However, the content of trace elements and the concentration of glucose were doubled (see Table 3).

Sodium citrate was also added to the feed solution as described in section “Materials and methods” and the pH of the feed solution was adjusted to 6.5 to prevent precipitation. No precipitation was observed in the feed during our experiments.

### Fed-batch cultivations

Fed-batch cultivations were performed in the Multifors bioreactor with the optimized low-chloride medium. With the Multifors, it is possible to operate two bioreactors in parallel. In the first batch (run A), medium VN6 (see Table 3) was used. The temperature was set to 30 °C. Figure 1 and Fig. 4 show that the osmolality of the standard medium VN1 is 1.08 Osmol kg^−1^ and therefore higher than that of the other media VN2 to VN10 (between 0.8 and 0.91 Osmol kg^−1^), because the total concentration of osmotically active particles is higher in the standard medium VN1. Therefore, in the second cultivation (run B), medium VN11 was used, in which the concentrations of Na_2_HPO_4_, Na_2_SO_4_, and sodium citrate were increased (see Table 3 for composition). The aim was to determine whether a higher salt concentration combined with a higher osmolality leads to better growth of the bacteria. These 2 cultivations were repeated with the media VN10 (run C) and VN12 (run D), where NH_4_Cl was replaced by (NH_4_)_2_SO_4_.

**Fig. 5:**
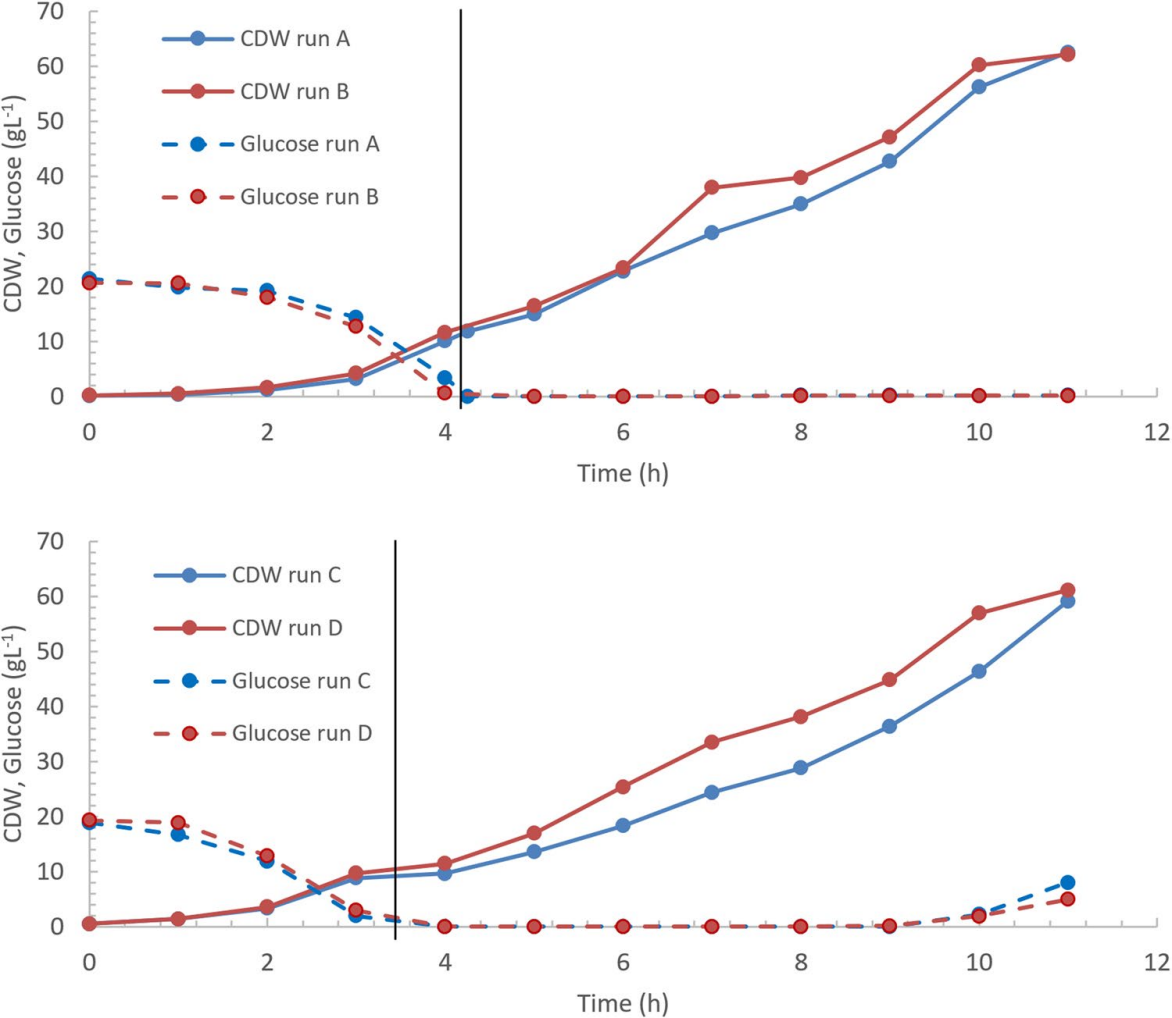
Time profiles of CDW and glucose during high-cell-density cultivations of *V. natriegens* in the optimized media VN6 (run A), VN11 (run B), VN10 (run C), and VN12 (run D). In run B and D, the sodium concentration was increased (see Table 3). After glucose limitation, the feed solution was fed exponentially indicated by the black vertical line

In Fig. 5, the time profiles of cultivation parameters for the fed-batch cultures of *V. natriegens* (denoted as run A, B, C, and D) are shown. The batch phase lasts approximately 4.2 h for run A and B and 3.5 h for run C and D until glucose was depleted. Biomass concentration increased exponentially while glucose concentration decreased during this phase. The maximum specific growth rate during the batch phase was between 0.9 h^−1^ in run B and 0.96 h^−1^ in run D.

**Fig. 6:**
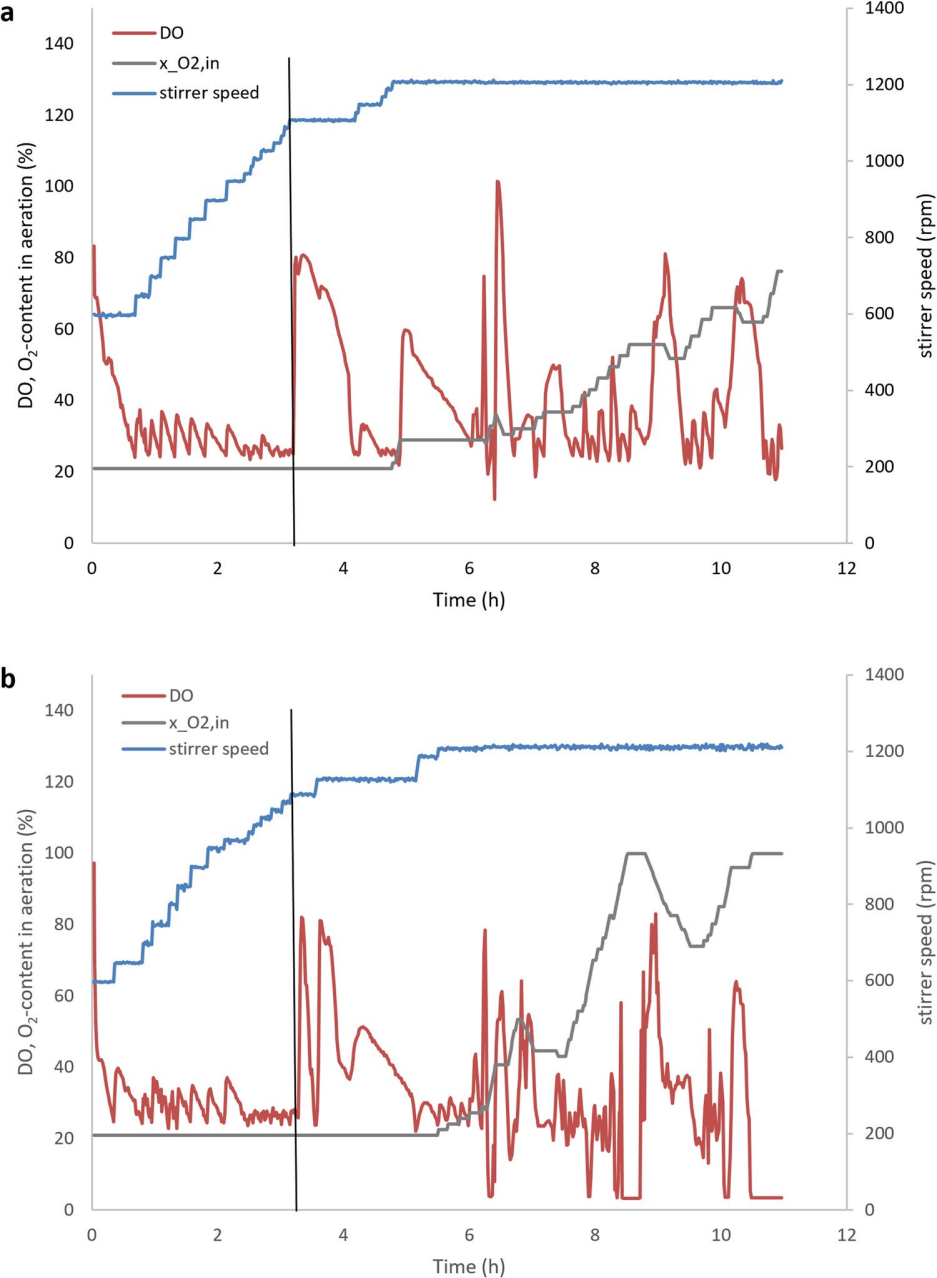
Time profiles of DO, O_2_-content in aeration, and stirrer speed during high-cell-density cultivations of *V. natriegens* in the optimized media VN10 (**a**, run C) and VN12 (**b**, run D). After glucose limitation, the feed solution was fed exponentially indicated by the black vertical line

Figure 6 shows the online parameters DO, stirrer speed, and O_2_-content in aeration for run C and D (see Supplementary Fig. S1 for the corresponding plots for run A and B). Glucose limitation leads to a sharp increase in DO (see Fig. 6 and Fig. S1). After glucose depletion, the exponential feeding was started with a set point for the specific growth rate of 0.3 h^−1^. This value was chosen mainly to prevent a limitation in the oxygen supply during the fed-batch phase. To avoid oxygen limitation, the reactor was operated temporarily at the maximum agitation rate of 1200 rpm, and the aeration gas was enriched with pure oxygen (see Fig. 6 and Fig. S1). During the fed-batch phase, the glucose concentration was initially below the detection limit of 90 mg L^−1^. After 10 h, the glucose concentration began to increase slightly in run C and D (see Fig. 5). The final biomass concentrations achieved in all 4 runs were in the same range (run A: 62.5 g L^−1^, run B: 62 g L^−1^, run C: 59 g L^−1^, run D: 61 g L^−1^).

**Fig. 7:**
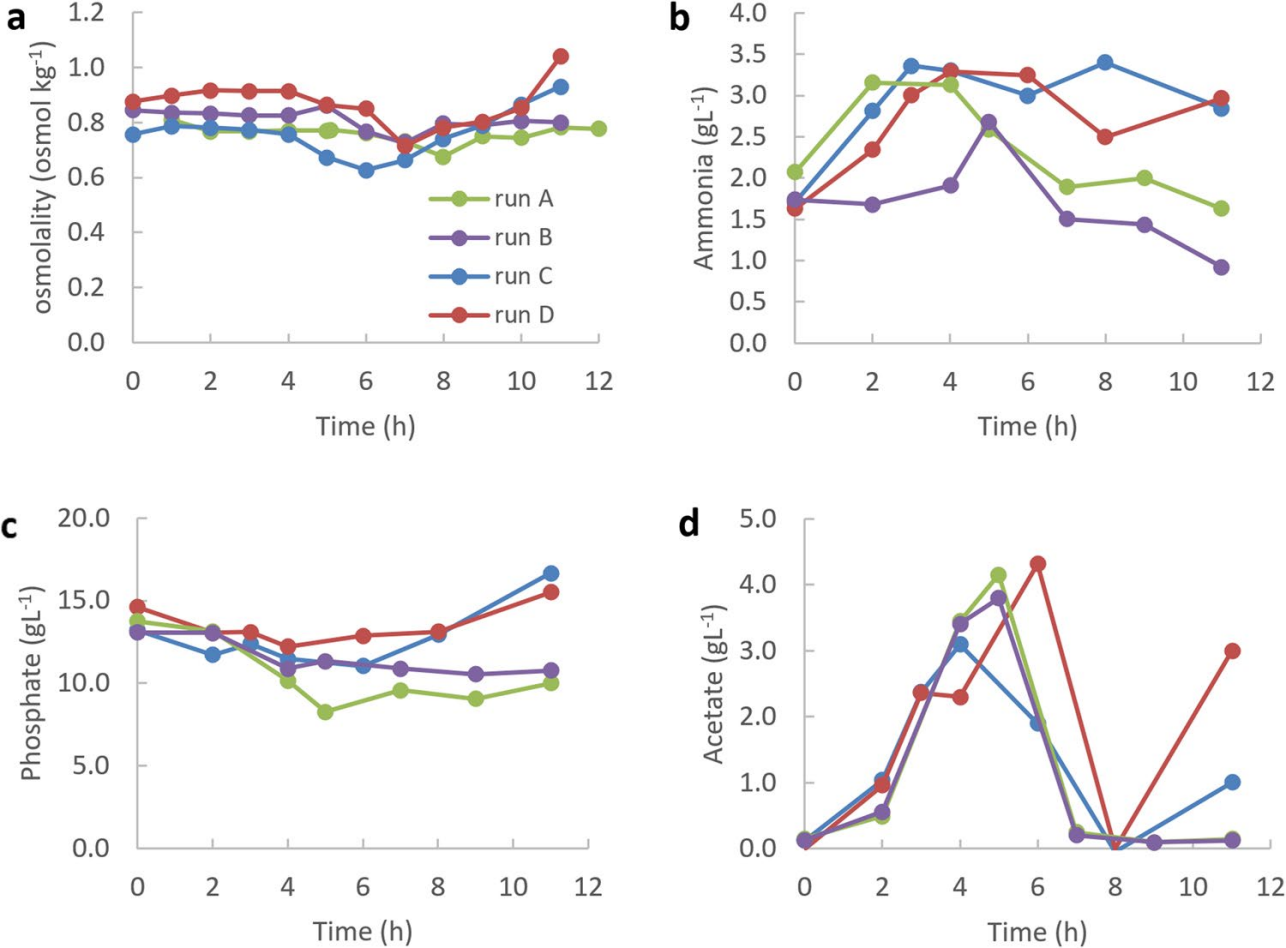
Time profiles of osmolality (**a**), ammonia (**b**), phosphate (**c**), and acetate (**d**) during high-cell-density cultivations of *V. natriegens* in the optimized media VN6 (run A), VN11 (run B), VN10 (run C), and VN12 (run D)

During the batch phase, the bacteria produced the by-product acetate (see Fig. 7). After glucose is completely consumed at the end of the batch phase, acetate is used up during the fed-batch phase. At the end of run C and D, its concentration begins to increase again as the glucose concentration increases.

The ammonium concentration in both reactors increases continuously during the batch phase due to the addition of ammonia water by pH control (see Fig. 7). As expected, the osmolality of run A and C was initially lower than that of run B and D. The osmolalities in run B and D are in the range of the osmolality in the standard medium VN1. The growth rate in run B and D decreases towards the end of cultivation, presumably due to temporary oxygen limitation (Fig. 6 and Fig. S1). However, the feed continues to be added exponentially. This causes substrates from the feed (e.g., phosphate) to accumulate in the bioreactor. This in turn leads to an increase in osmolality.

## Discussion

The majority of chemically defined media used for the cultivation of *V. natriegens* contain high concentration of NaCl (Becker et al. 2019; Hoff et al. 2020; Hoffart et al. 2017; Lee et al. 2019; Thiele et al. 2021). This may lead to chloride-induced corrosion processes of metal surfaces in bioreactors. Therefore, we developed a chemically defined medium that does not contain NaCl. NaCl can be completely replaced by a mixture of Na_2_HPO_4_, Na_2_SO_4_, and sodium citrate. In this new medium, *V. natriegens* grows with a specific growth rate of up to 0.96 h^−1^ at 30 °C. This is comparable to the growth rates in the standard medium VN1. Thiele et al. (2021) report growth rates in a chemically defined medium with NaCl of 0.79 h^−1^ at 30 °C and 1.36 h^−1^ at 37 °C.

Hoffart et al. (2017) also designed a defined medium containing only traces of chloride but sufficient sodium ions to maintain the high growth rate of *V. natriegens*. The chloride concentration in the optimized medium VN10 and VN12 was reduced from 12.65 g L^−1^ to 10.3 mg L^−1^ or 10.3 ppm which is uncritical to stainless steels used in industrial bioreactors. For 316 stainless steel, the water chloride limit for crevice corrosion is about 1000 ppm (Junker 2009). The safe high level of chloride is considered to be about 150 ppm for continuous exposure of 316 stainless steel, particularly at high temperatures such as could occur during sterilization (Chisti 1992).

The use of citrate in our medium is important to prevent possible precipitation. By substituting sodium chloride with sodium hydrogen phosphate and sodium sulfate without the use of sodium citrate, precipitates in the form of AMP were observed. In defined media, the appearance of AMP crystals connected with a loss of magnesium, ammonium, and phosphate ions is a common phenomenon (Shiloach and Fass 2005; Pirt 1975). AMP is sparingly soluble in water and the solubility is pH dependent. As the pH increases, the solubility decreases. At a pH of 10.3, the solubility is lowest (Ohlinger et al. 1998). Since in our work, NaCl was partially replaced by sodium phosphate, precipitation of AMP in the modified media may increase due to the increased PO_4_^3−^ ion concentration. Low pH values are favorable to prevent precipitations but at a pH value of 6.5 or below, the growth of *V. natriegens* was significantly reduced. In complex media, growth is optimal at pH 7.5, and at lower pH values, the growth rate is reduced (Payne et al. 1961). In our experiments, the bacteria grew at pH 6.75 comparably to pH 7.

Accurate determination of AMP solubility requires consideration of ionic strength effects on effective ion concentrations in the aqueous system and inclusion of magnesium complexes in the analysis (Ohlinger et al. 1998). Ionic strength affects precipitation potential because the electrostatic interactions of ions in solution reduce their activity, or effective concentration, thereby reducing AMP precipitation potential. Magnesium phosphate complex formation reduces the concentrations of Mg^2+^ and PO4^3−^ ions available for AMP formation (Ohlinger et al. 1998). The use of citrate in our media probably prevented precipitation due to the formation of magnesium citrate complexes. The complexed magnesium ions were nevertheless bioavailable to the cells. This confirms sodium citrate as a potentially important ingredient of the medium to prevent precipitations. It can even be used as a carbon source for bacteria for *V. natriegens* (Austin et al. 1978).

In the media with EDTA as a complexing agent, the growth of *V. natriegens* was significantly reduced. The inhibitory effect of EDTA on growth has also been described for other microorganisms (Root et al. 1988). The chelator forms extremely firm soluble complexes with divalent and trivalent metals (Root et al. 1988), and it is most likely that in our experiments, the concentration of one or more metal ions necessary for bacterial growth was reduced to below a critical level.

To prevent precipitation, media components should be sterilized separately and mixed only afterwards. Pirt (1975) also describes the phenomenon that precipitation may not occur until several hours after the solution has been prepared. Thus, the preparation of the medium, especially the addition of the sterile MgSO_4_ stock solution, should be done just before inoculation. This practice was implemented in the preparation of the media in this work.

In fed-batch cultivations, using this newly developed low-chloride medium biomass conc. up to 62 g L^−1^ at 30 °C could be achieved. Biomass concentrations of 55 g L^−1^ were described in fed-batch cultivations in a chemically defined medium containing NaCl at 30 °C (Thiele et al. 2021). The maximum specific growth rate during the batch phase was 0.96 h^−1^. In Thiele et al., 0.79 h^−1^ were reached at 30 °C in a minimal medium containing NaCl.

The demand for ammonium during cultivation is covered by ammonium sulfate in the basic medium and by the addition of ammonia water through pH control. The ammonium concentration drops slightly during the fed-batch phase, but is never limiting (Fig. 7). This shows that the supply of the only nitrogen source ammonium is sufficient.

The specific growth rate of the exponential feeding during the fed-batch phase was set to 0.3 h^−1^. This is approximately 30% of the maximum specific growth rate. This value was chosen mainly to avoid oxygen limitation during the fed-batch phase and to prevent the formation of overflow metabolites such as acetate. The set point value of specific growth can be increased by using a bioreactor with higher power inputs or higher volumetric oxygen transfer coefficient (k_L_a) values, respectively. This could lead to higher space-time yields. Another strategy often used in fed-batch cultivations is to switch to a constant feed rate after the oxygen transfer rate (OTR) reaches its maximum during exponential feeding. However, Thiele et al. (2021) showed that upon switching from exponential to constant glucose-feeding, cell death was induced.

An increase in specific growth rate might also induce overflow metabolism and the production of overflow metabolites such as acetate. In *E. coli* cultivations, the formation of growth inhibitory by-products such as acetate has to be minimized to be able to reach high-cell densities (Riesenberg 1991; Biener et al. 2010). During the batch phase, acetate was produced up to a concentration 4.3 g L^−1^. In the subsequent fed-batch phase, acetate concentration decreased to 0 g L^−1^. This indicates that the specific growth rate for the exponential feeding may be increased. In this context, a possible inhibitory influence of acetate on growth should also be further investigated.

In run A and C, NaCl was replaced by the 3 sodium salts under the premise that the total concentration of sodium was unchanged. In run B and D, the concentration of the 3 sodium salts was increased to such an extent that the osmolalities were in the range to the values of the standard medium VN1. Comparing the measured data to run A and C shows that the specific growth rate during the batch phase was slightly higher in run B and D. This trend continued in the fed-batch phase, despite temporary oxygen limitation towards the end of cultivation. These results suggest that both total sodium concentration and osmolality play important roles in achieving higher cell densities.

In summary, the chemically defined low-chloride media developed can realize high biomass yields while preventing corrosion of the bioreactor steel. This will allow the strain to reach its full potential for industrial applications in the future.

### Supplementary information

ESM 1 (PDF 191 kb)

## Data Availability

The datasets generated during and/or analyzed during the current study are available from the corresponding author on reasonable request.
