# Supplementary material for: High-cell-density cultivation of Vibrio natriegens in a low-chloride chemically defined medium
Source: Appl Microbiol Biotechnol. 2023 Sep 23;107(23):7043–54. doi: 10.1007/s00253-023-12799-4 (PMC10638117; doi:10.1007/s00253-023-12799-4)
Supplement: Supplementary file 1 — (PDF 191 kb) [file 253_2023_12799_MOESM1_ESM.pdf]

Supplementary Information

High cell density cultivation of *Vibrio natriegens* in a low-chloride chemically defined medium

Journal Name: Applied Microbiology and Biotechnology

Richard Biener*, Thomas Horn, Alexander Komitakis, Ines Schendel, Leon König, Anna Hauenstein, Alina Ludl, Andrea Speidel, Svenja Schmid, Julian Weißer, Max Broßmann, Sofia Kern, Max Kronmüller, Sonja Vierkorn, Lennart Suckow, Arthur Braun

University of Applied Sciences Esslingen, Faculty of Science, Energy and Building Services

Kanalstraße 33, 73728 Esslingen, Germany

*corresponding author

e-mail address of corresponding author: richard.biener@hs-esslingen.de

ORCID of corresponding author: 0009-0000-4015-0095

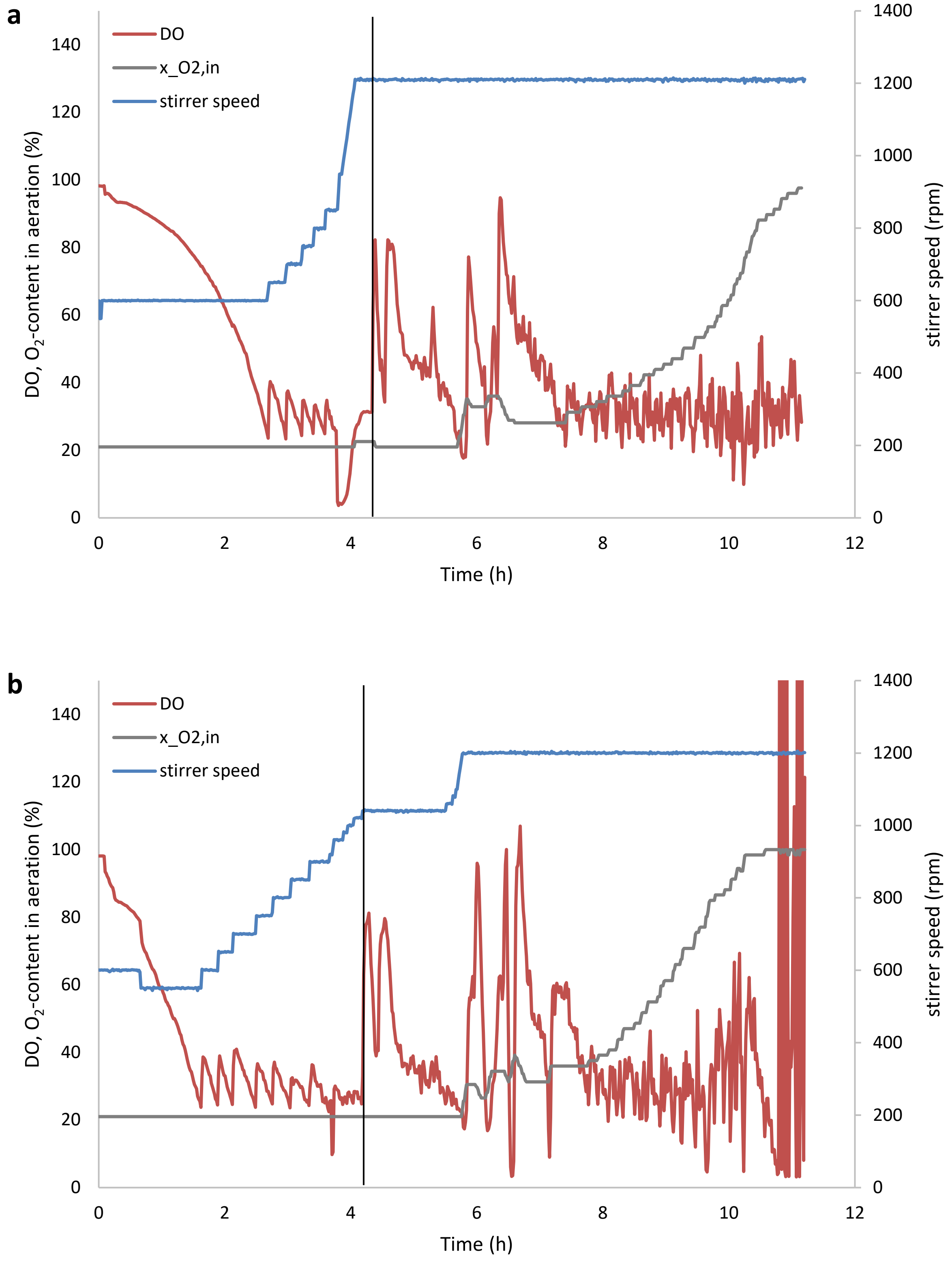

Fig. S1 Time profiles of DO, O_2-content in aeration and stirrer speed during high cell density cultivations of *V. natriegens* in the optimized media VN6 (a, run A) and VN11 (b, run B). After glucose limitation the feed solution was fed exponentially indicated by the black vertical line.
